# King's College Hospital

**Published:** 1905-05-27

**Authors:** 


### KING'S COLLEGE HOSPITAL.

### FESTIVAL DINNER.

Lord Roberts presided at the festival dinner of King's College Hospital, which was held last week at the Hôtel Cecil. The result of the appeal made on this occasion was that a sum of about £1,700 was added to the funds of the hospital.

The Chairman, after the loyal toasts had been honoured, proposed "Success to King's College Hospital." Recently, he said, he had paid a visit to the hospital, and, as far as he could judge—and he had visited many hospitals under various conditions in all parts of the world—everything was as it should be. King's College Hospital had been a pioneer in the matter of antiseptic surgery, for it was at that hospital that Lord Lister had mainly carried on the experiments which led to his great discoveries in antiseptic surgery. He (the speaker) could speak from experience of the value of antiseptic surgery, as he well remembered the conditions which prevailed before antiseptic surgery was practised. In the early months of the siege of Delhi, for instance, nearly every amputation had meant a death. That could well be understood, for the men were crowded together, and what that meant in a very hot climate, where the flies were a perfect plague, his hearers could realise. He had often since thought what a mercy it would have been for them all if they had had a Lord Lister 50 years earlier.

Lord Methuen, who for the last year and a half has been the Chairman of the Committee of Management, responded. As a soldier he had had a great deal to do with hospital work, such as he was at the present time carrying out, because in his command it was his duty to see to the comfort of those in his own military hospitals. He had had, therefore, a very good chance of comparing the manner in which the work was carried out in military hospitals as compared with the work in civilian hospitals. He might say at the outset that whether it was the way in which the treatment was carried out by their medical officers, by their sisters, or by their nurses, in the military hospitals, or the amount of space that was given to them, in his opinion military hospitals compared quite favourably with civilian hospitals. The Chairman had alluded to the generosity of London towards its sick poor. He (the speaker) thought it was quite a fair comparison to make to compare the requirements of the Kingdom and of the Army with those of the large towns of England and their hospitals. As this Empire increased so did the requirements of the Empire and the Army, and they knew perfectly well that unless the Kingdom could voluntarily support the Army, which was larger now than ever, that there was but one end, and that was annihilation. So it was with our hospitals. The requirements of the hospitals were far greater now than they had been at any former time, and those requirements would go on increasing. Unless the voluntary system was given the support it wanted, there was but one end, and that was that the hospitals would have to go on the rates, and unless the people in London came forward and supported the great hospitals—and the demands of St. Bartholomew's, of King's College, and Guy's, were greater than ever—the time might come when those hospitals would come on the rates. They might then take it that the expenses of the hospitals would increase, whilst the general management would certainly not be more satisfactory than it was at the present time. (Hear, hear.) He therefore thought he was justified in urging every one to recollect that the men who were doing the work for the hospitals were not the men whose pockets were the fullest, and that those people who had made their money by the work of the poor in London were the persons who should put their hands deeply into their pockets for the support of the poorer classes of the metropolis through whom they had made their wealth. (Applause.)

In replying for the "Medical and Nursing Staff of the Hospital," Mr. Watson Cheyne said there was another reason for moving the hospital besides that which the Chairman had mentioned, viz., the migration of the poor from the neighbourhood of the present hospital to the south side of the Thames, and that was that if they had not moved to another site they would have had to face the problem of pulling down the old hospital and building a modern one on the old site. This would have entailed a considerable diminution in the number of beds provided.

Other toasts were proposed, and the proceedings terminated with a vote of thanks to the Chairman.

